# Impact of domestic travel restrictions on transmission of COVID-19 infection using public transportation network approach

**DOI:** 10.1038/s41598-021-81806-3

**Published:** 2021-02-04

**Authors:** Yayoi Murano, Ryo Ueno, Shoi Shi, Takayuki Kawashima, Yuta Tanoue, Shiori Tanaka, Shuhei Nomura, Hiromichi Shoji, Toshiaki Shimizu, Huy Nguyen, Hiroaki Miyata, Stuart Gilmour, Daisuke Yoneoka

**Affiliations:** 1 Division of Biostatistics and Bioinformatics, Graduate School of Public Health, St. Luke’s International University, OMURA Susumu and Mieko Memorial, St. Luke’s Center for Clinical Academia, 5th Floor 3-6-2 Tsukiji, Chuo-ku, Tokyo, 104-0045 Japan; 2 Toshima Hospital, 33-1 Sakaecho, Itabashi-ku, Tokyo, Japan; 3 Department of Paediatrics and Adolescents, Juntendo University, 2-1-1 Hongo Bunkyo-ku, Tokyo, Japan; 4 The Australian and New Zealand Intensive Care Research Centre, 553 St Kilda Road, Melbourne, VIC 3004 Australia; 5 Graduate School of Medicine, The University of Tokyo, 7-3-1 Hongo Bunkyo-ku, Tokyo, Japan; 6 Department of Mathematical and Computing Science, Tokyo Institute of Technology, 2-12-1 Ookayama Meguro-ku, Tokyo, Japan; 7 Institute for Business and Finance, Waseda University, 1-6-1 Nishi-Waseda, Shinjuku-ku, Tokyo, Japan; 8 Epidemiology and Prevention Group, Center for Public Health Sciences, National Cancer Center, 5-1-1 Tsukiji, Chuo-ku, Tokyo, Japan; 9 Department of Health Policy and Management, School of Medicine, Keio University, Endo, Fujisawa-city, Kanagawa 4411 Japan

**Keywords:** Diseases, Viral infection

## Abstract

The international spread of COVID-19 infection has attracted global attention, but the impact of local or domestic travel restriction on public transportation network remains unclear. Passenger volume data for the domestic public transportation network in Japan and the time at which the first confirmed COVID-19 case was observed in each prefecture were extracted from public data sources. A survival approach in which a hazard was modeled as a function of the closeness centrality on the network was utilized to estimate the risk of importation of COVID-19 in each prefecture. A total of 46 prefectures with imported cases were identified. Hypothetical scenario analyses indicated that both strategies of locking down the metropolitan areas and restricting domestic airline travel would be equally effective in reducing the risk of importation of COVID-19. While caution is necessary that the data were limited to June 2020 when the pandemic was in its initial stage and that no other virus spreading routes have been considered, domestic travel restrictions were effective to prevent the spread of COVID-19 on public transportation network in Japan. Instead of lockdown that might seriously damage the economy, milder travel restrictions could have the similar impact on controlling the domestic transmission of COVID-19.

## Introduction

As of July 3, 2020, 516,210 deaths out of 1,069,4288 laboratory-confirmed infectious cases of novel coronavirus (COVID-19) were reported globally^1^. The rapid growth of cases worldwide, including in Asian and European countries and the United States, was accelerated by global and domestic human movements. Most of the current strategies to reduce the risk of COVID-19 transmission, such as social distancing, case isolation and contact tracing, are based on restricting human–human interactions^2^. In particular, one of the popular strategies to control human–human interaction worldwide is a travel restriction between countries. However, previous studies report that global travel restrictions at national borders has only limited usefulness for infection control^3,4^, and moreover, the restriction would damage global/local economies^5,6^. Therefore, it may be more efficient to control the COVID-19 spread at the local or domestic level rather than to attempt to control the epidemic at the national borders^4,7–9^.

Japan has experienced comparatively slow growth in the number of COVID-19 cases and low death rate among the G7 nations^1,10^: on 3rd July 2020, 18,723 people were infected and 974 died from COVID-19. Moreover, Japan did not enforce emergency lockdown with penalties as was done in many countries in Europe or the United States^11^. Instead, voluntary restraint on non-emergency and/or non-urgent travel were publicly requested by the government. This means that control of the domestic infections was essentially driven by the voluntary efforts of Japanese residents^12,13^. It is known that lockdowns are effective for containment or suppression of COVID-19 in the short term^11^, but they can have significant negative economic, social, mental health and other outcomes. Moreover, lockdowns need to be carefully calibrated in length and severity to balance economic and public health impact, while minimizing the risk of a second or third wave of COVID-19. Several mathematical modeling techniques have been developed to evaluate the impact of travel restriction that is induced by lockdowns or other policies with penalty^3,7,14–18^: Colizza et al.^15^ proposed a new stochastic meta-population model that combined person-trip records and census data in 220 countries to predict the spread of SARS internationally. Shi et al.^7^ and Otsuki and Nishiura^3^, whose methodological approach we followed, estimated the risk reduction due to travel restrictions using a hazard-based modeling technique, concluding that the restrictions were not effective enough to prevent the global spread of COVID-19 and Ebola virus disease, respectively. A comprehensive overview of the relationship between transportation network and spread of infectious diseases can be found in Barabasi^19^ and Tatem et al.^20^. However, there are still few studies that examine the effect of local or domestic travel restrictions, such as restrictions on car, train, ship and airline networks, on the COVID-19 epidemic^4,21^.

In this study, we estimated the impact of domestic travel restrictions on the spread of COVID-19 in Japan using a hazard-based model and the concept of network (closeness) centrality on the public transportation. To examine the impact of lockdown on the transmission of COVID-19 on the network, we hypothetically simulated the lockdown situation to restrict domestic travel between metropolitan areas, and then compared with other plausible scenarios.

## Results

### Public transportation network

**Figure 1:**
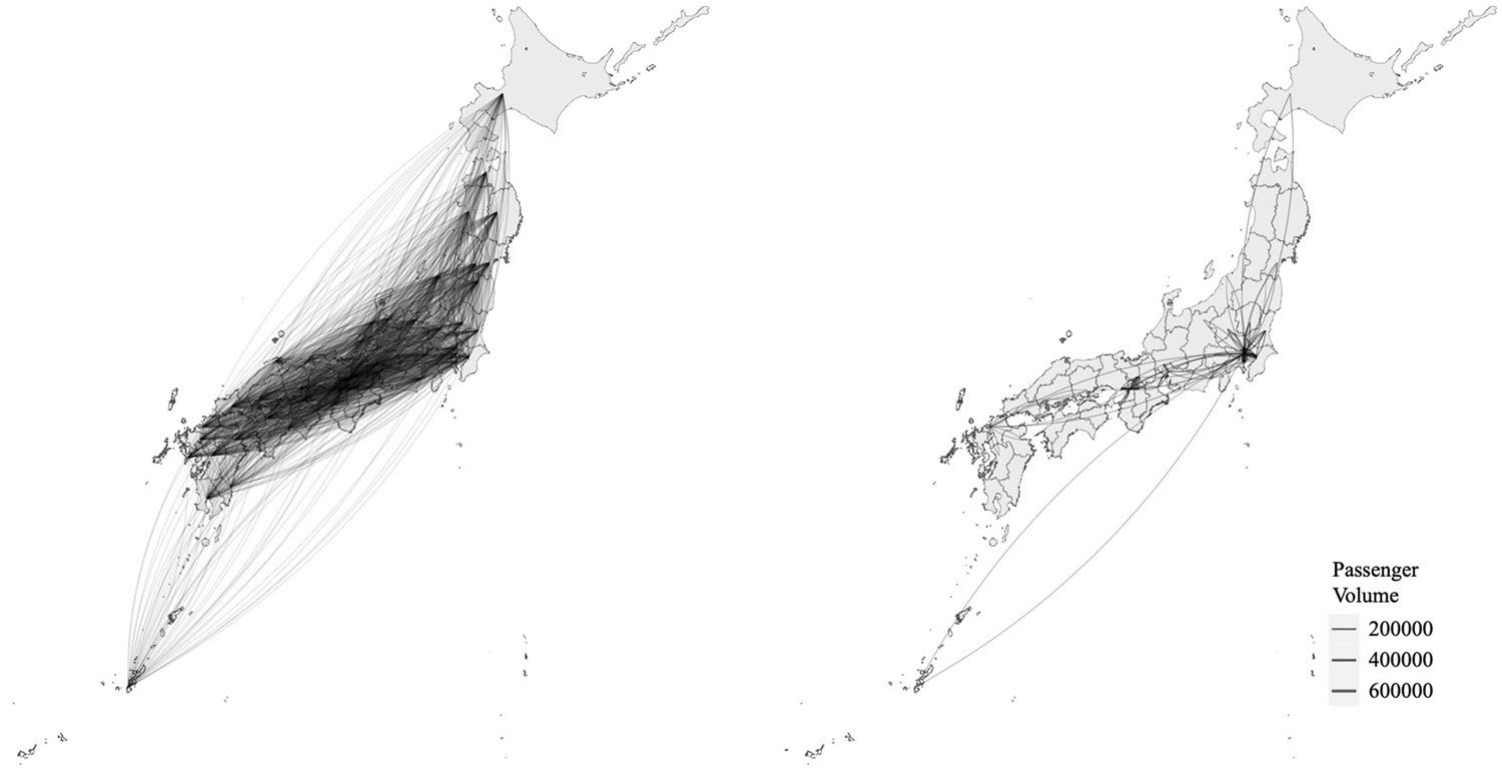
Public transportation network in Japan (Left) and top 5% most populous passenger travels (Right).

Figure 1 shows the entire public transportation network (Left) and top 5% populous passenger travels (i.e., the edges constituting the top 5% passenger volumes) in Japan. The list of prefectures that had experienced the importation of COVID-19 and the associated covariate information are shown in Supplementary Table. A total of 46 prefectures with imported COVID-19 cases have been identified (except Iwate prefecture, which corresponds to (D) at the end of the study period). Thirty-five prefectures imported COVID-19 before March 25 (i.e., corresponding to (A)), 8 prefectures imported COVID-19 between March 25 and April 7 (i.e., corresponding to (B)), and 3 prefectures imported COVID-19 after April 7 (i.e., corresponding to (C)). The arrival time ranged from 16 to 86 days (mean: 52.5, standard deviation: 18, median: 50 days) since the first case was identified on January 15.

### Risk assessment

**Figure 2:**
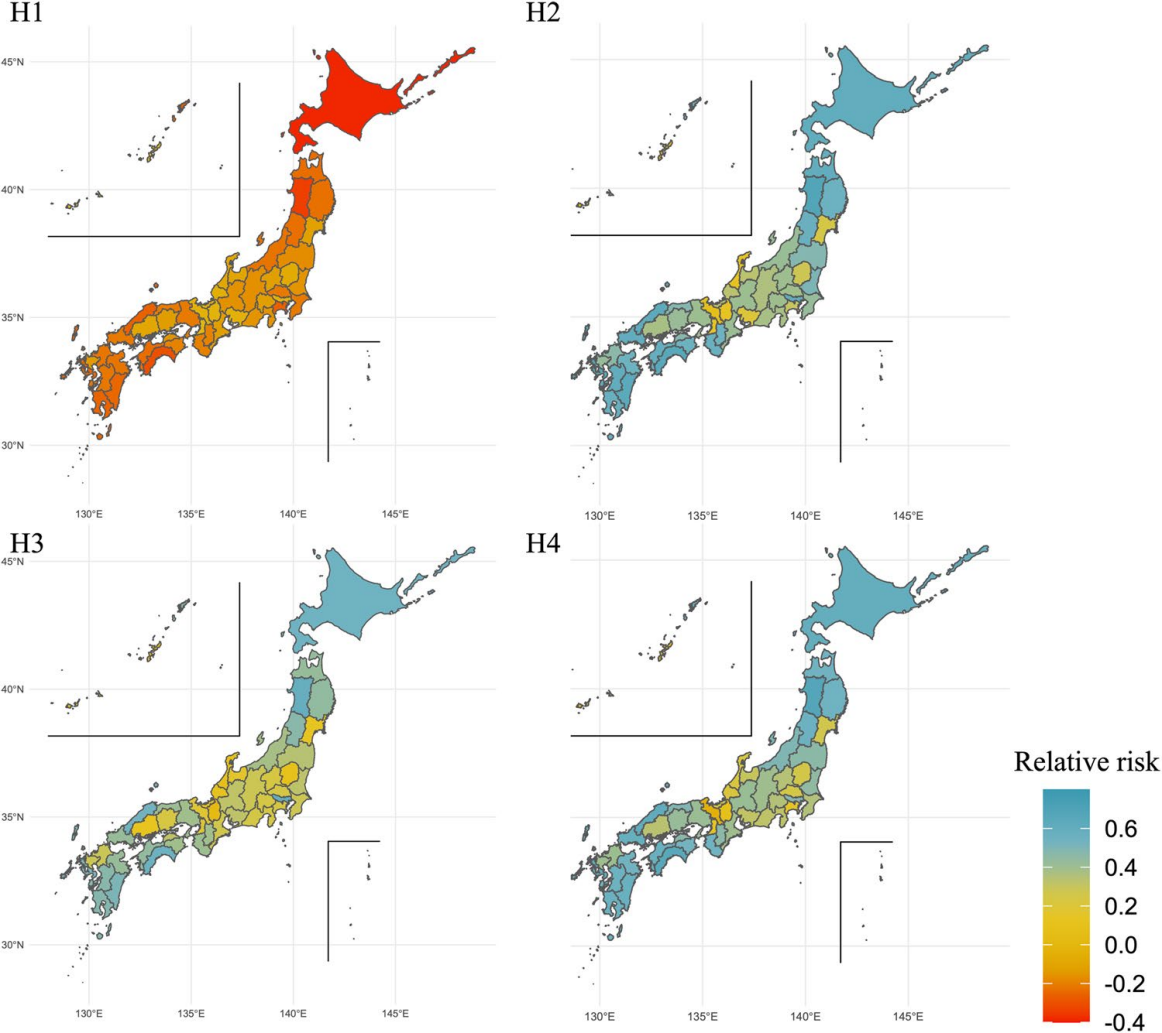
Estimated relative risks by hypothetical scenarios: (H1) no travel restriction scenario, (H2) lockdown-based scenario, (H3) delete the top 10 populous passenger volumes edges scenario, and (H4) restriction of airplane travel scenario.

Table 1 and Fig. 2 show the estimated risk reduction due to the domestic travel restrictions and the associated change of closeness centrality on public transportation networks in each prefecture. Figure 2 (H1) shows the estimated relative risk reduction under the assumption of (H1): no travel restriction had taken place. The median (25% and 75% percentiles) of estimated relative risk reduction were − 0.191 (− 0.225, − 0.116), indicating that all prefectures would have increased the risk of importation of COVID-19 by (around) 19% if no travel restriction had been imposed. In contrast, when the hypothetical travel restrictions would have taken place in the scenarios under H2–H4, positive risk reductions were observed in most prefectures, especially in Northern and Southern parts of Japan such as Hokkaido and Kagoshima prefectures and the neighborhood prefectures of metropolitan areas such as Saitama and Chiba prefectures, which are located next to Tokyo prefecture. Figure 2 (H2–H4) shows the estimated relative risk reduction under the assumption of (H2–H4). The median (25% and 75% percentiles) estimated relative risk reductions were 0.481 (0.389, 0.520), 0.342 (0.273, 0.384), and 0.456 (0.372, 0.478) for H2–H4, respectively, indicating that H2 (i.e., the lockdown-based scenario) has the strongest impact on the reduction of the risk of importing COVID-19 in each prefecture: by locking down Tokyo during P2 and the 7 prefectures during P3, the risk would have been reduced by (around) 48.1% compared with the Observed risk. However, note that the magnitude of the risk reduction under H2 was relatively similar to that under H4, suggesting that it is possible to obtain similar risk reduction without implementing the strict lockdown. Risk reductions under H2–H4 had similar geographical tendency: northern and southern parts of Japan such as Hokkaido, Miyagi, Nagasaki and Miyazaki prefectures, and neighborhood prefectures of metropolitan area such as Ibaraki and Chiba prefectures, saw the largest risk reductions under all scenarios.

**Table 1:** Estimated risk and relative risks by hypothetical scenarios.

Prefecture	Estimated risk					Estimated relative risk			
	Scenarios					Scenarios			
	Observed	H1	H2	H3	H4	H1	H2	H3	H4
HOKKAIDO	0.4567	0.6323	0.1980	0.2318	0.2057	− 0.3847	**0.5665**	**0.4924**	**0.5496**
AOMORI	0.6831	0.8428	0.3093	0.4482	0.3647	− 0.2339	**0.5473**	**0.3439**	**0.4661**
IWATE	0.6996	0.8590	0.3502	0.4406	0.3628	− 0.2278	**0.4994**	**0.3702**	**0.4815**
MIYAGI	0.8636	0.9600	0.5251	0.5995	0.5100	− **0.1116**	0.3919	0.3058	0.4095
AKITA	0.5096	0.6845	0.2362	0.2997	0.2385	− 0.3432	**0.5366**	**0.4119**	**0.5320**
YAMAGATA	0.6953	0.8556	0.3454	0.4285	0.3598	− 0.2305	**0.5032**	**0.3837**	**0.4826**
FUKUSHIMA	0.7813	0.9143	0.4053	0.5235	0.4254	− **0.1703**	0.4813	0.3299	0.4556
IBARAKI	0.8037	0.9264	0.3918	0.5351	0.4365	− **0.1527**	**0.5125**	0.3341	**0.4568**
TOCHIGI	0.9182	0.9822	0.5645	0.7080	0.5854	− **0.0698**	0.3852	0.2289	0.3624
GUNMA	0.8488	0.9516	0.4819	0.6155	0.5255	− **0.1211**	0.4323	0.2749	0.3809
SAITAMA	0.7227	0.8746	0.3386	0.4204	0.4167	− 0.2103	**0.5315**	**0.4182**	0.4234
CHIBA	0.7257	0.8772	0.3464	0.4324	0.3914	− 0.2089	**0.5226**	**0.4041**	**0.4606**
TOKYO	0.9558	0.9962	0.3825	0.3950	0.5047	− **0.0422**	**0.5998**	**0.5868**	**0.4720**
KANAGAWA	0.6344	0.7983	0.3126	0.3440	0.3841	− 0.2584	**0.5072**	**0.4577**	0.3945
NIIGATA	0.6972	0.8519	0.4042	0.4299	0.3487	− 0.2219	0.4203	**0.3835**	**0.4999**
TOYAMA	0.8787	0.9666	0.5728	0.7178	0.6015	− **0.1000**	0.3481	0.1831	0.3155
ISHIKAWA	0.8853	0.9651	0.6520	0.6728	0.5729	− **0.0901**	0.2636	0.2400	0.3529
FUKUI	0.9400	0.9893	0.6254	0.8269	0.7334	− **0.0524**	0.3347	0.1203	0.2198
YAMANASHI	0.8502	0.9547	0.4866	0.6107	0.5233	− **0.1230**	0.4277	0.2817	0.3845
NAGANO	0.7874	0.9164	0.5286	0.5966	0.5008	− **0.1639**	0.3286	0.2423	0.3640
GIFU	0.7838	0.9085	0.4592	0.5240	0.4488	− **0.1592**	0.4141	0.3314	0.4274
SHIZUOKA	0.8051	0.9278	0.5149	0.6165	0.5628	− **0.1525**	0.3604	0.2342	0.3010
AICHI	0.9577	0.9938	0.7276	0.6219	0.6298	− **0.0376**	0.2403	**0.3506**	0.3424
MIE	0.8459	0.9524	0.4805	0.6003	0.4970	− **0.1259**	0.4320	0.2904	0.4125
SHIGA	0.9717	0.9964	0.7230	0.8174	0.7589	− **0.0254**	0.2560	0.1588	0.2190
KYOTO	0.9641	0.9952	0.7974	0.7036	0.8986	− **0.0322**	0.1730	0.2702	0.0680
OSAKA	0.9978	0.9999	0.8509	0.8014	0.8520	− **0.0021**	0.1472	0.1968	0.1462
HYOGO	0.7204	0.8708	0.3898	0.3948	0.3823	− 0.2087	0.4590	**0.4520**	**0.4693**
NARA	0.7540	0.8984	0.3513	0.4671	0.4127	− 0.1916	**0.5341**	**0.3804**	0.4526
WAKAYAMA	0.7513	0.8984	0.3681	0.5065	0.3944	− 0.1959	**0.5100**	0.3258	**0.4750**
TOTTORI	0.7225	0.8729	0.3988	0.4535	0.3887	− 0.2081	0.4481	**0.3724**	**0.4620**
SHIMANE	0.6488	0.8154	0.3182	0.4008	0.3305	− 0.2566	**0.5096**	**0.3823**	**0.4906**
OKAYAMA	0.8291	0.9383	0.4954	0.6511	0.4916	− **0.1317**	0.4025	0.2147	0.4071
HIROSHIMA	0.8904	0.9707	0.5475	0.7594	0.5842	− **0.0902**	0.3851	0.1471	0.3439
YAMAGUCHI	0.7082	0.8572	0.3478	0.5039	0.3679	− 0.2103	**0.5089**	0.2886	**0.4805**
TOKUSHIMA	0.7485	0.8929	0.3707	0.4925	0.3842	− 0.1929	**0.5048**	0.3420	**0.4867**
KAGAWA	0.8047	0.9270	0.4402	0.5719	0.4472	− **0.1520**	0.4529	0.2893	0.4443
EHIME	0.6869	0.8480	0.3224	0.4408	0.3408	− 0.2344	**0.5307**	**0.3584**	**0.5039**
KOCHI	0.5918	0.7679	0.2745	0.3455	0.2722	− 0.2975	**0.5362**	**0.4163**	**0.5401**
FUKUOKA	0.7091	0.8648	0.3421	0.4552	0.3820	− 0.2194	**0.5177**	**0.3581**	**0.4613**
SAGA	0.8283	0.9439	0.4709	0.5867	0.4955	− **0.1395**	0.4315	0.2917	0.4018
NAGASAKI	0.6743	0.8410	0.2905	0.4082	0.3297	− 0.2471	**0.5692**	**0.3947**	**0.5110**
KUMAMOTO	0.7099	0.8620	0.3279	0.4395	0.3764	− 0.2142	**0.5381**	**0.3809**	**0.4699**
OITA	0.7017	0.8567	0.3597	0.4540	0.3697	− 0.2209	**0.4874**	**0.3530**	**0.4731**
MIYAZAKI	0.6723	0.8373	0.2962	0.4056	0.3521	− 0.2454	**0.5595**	**0.3968**	**0.4763**
KAGOSHIMA	0.6669	0.8341	0.3244	0.3964	0.3377	− 0.2507	**0.5136**	**0.4057**	**0.4937**
OKINAWA	1.0000	1.0000	0.9991	1.0000	0.9966	**0.0000**	0.0009	0.0000	0.0034

Bold indicate relative risk values greater than the median value within each scenario.

## Discussion

This is the first study to estimate the relative risk reduction of domestic dissemination of COVID-19 in Japan due to travel restrictions on the public transportation network, using a hazard-based model and the idea of network centrality. The degree of travel restriction was assumed to be an 80% reduction in the passenger volume between Tokyo and other prefectures during March 24 to April 7, 2020, and between the 7 prefectures (Tokyo, Kanagawa, Chiba, Saitama, Osaka, Hyogo, and Fukuoka prefectures) and other prefectures after April 7, 2020. If no travel restriction would have taken place (Fig. 2, H1), all prefectures showed an increase in the relative risk, suggesting that it was better than doing nothing. To examine the hypothetical situations where other travel restriction would have taken place during the study period, we changed the degree of reduction in passenger volume, resulting in a change in centrality of each prefecture on the public transportation network. In all scenarios, we observed a volume-dependent reduction in relative risk (around 35–48% reductions) in most prefectures, especially in the most Northern and Southern (local) areas of Japan and the neighborhood prefectures of metropolitan areas (Fig. 2, H2–H4). This suggests that the degree of passenger volume has a large impact on the risk reduction and the optimal size of volume reduction may highly depend on the domestic network. In particular, the lockdown-based policy to control the passenger volumes (i.e., H2) would have similar impact on the risk reduction to a policy that restricts airline travel only (i.e., H4). This suggests that, instead of strictly locking down the metorpolitan areas that might seriously damage economy, milder travel restrictions (with associated compensation for economic damage) could have a similar impact on domestic transmission of COVID-19 without devastating economic damages. Airline restrition under H4 is a plausible and realistic strategy that allows continued daily life by maintaining the volume of essential cargo while minimizing the transmission of the infection because airplanes account only for 0.2% of the total domestic cargo while cars and ships account for 63.9% and 32.0%, respectively^22^. In addition, they are easily restricted compared to other transportations by airport closure. Based on these results, it can be concluded that the domestic travel restrictions based on passenger reductions would make a significant contribution to the prevention of virus importation within a country. Especially, this study showed the biggest impact of travel restriction is in the far north and south part of Japan, and thus in one of those areas such as Hokkaido prefecture, where had an outbreak^23,24^, earlier and more aggressive travel restrictions might have prevented the outbreak. Finally, our study proposes a framework for evaluating the impact of travel restrictions using a hazard function with the network closeness measure. This framework can be adapted to any nations given data on the date of onset for each region and data on regional mobility. Where travel restrictions were introduced in conjunction with other interventions such as lockdowns or non-pharmaceutical interventions, or where travel restrictions were applied at different times within regions (such as the EU) or nations (such as the USA), our method offers a tool to determine their effectiveness. Travel restrictions have been a controversial strategy since the West African Ebola epidemic, and it is important to evaluate their impact in order to improve global and national responses to future emerging disease pandemics. We welcome re-evaluation in other settings.

Our study has several limitations. Domestic travel is not only the driving force of virus spread. The international importation of COVID-19 from outside the country could potentially drive domestic virus spread, especially in areas where countries are adjacent to each other, such as European countries. However, since almost all countries are now closing their national borders, our results that focused on only domestic travel have significant implication. Another limitation is that our data on the date of first COVID-19 case might include a ‘reporting delay’. Since we modeled the hazard functions for the probability of the first case for each prefecture, the results should be biased due to the delay. However, it is notable that the delayed reports are gradually updated to correct the information in Japan, and thus the bias might be minimal. In addition, as with previous studies, the infected individual in this study was assumed to be randomly selected and move around all of Japan. Therefore, if the infected individuals had some characteristics, such as a preference for a particular travel route from one prefecture to other prefectures, our results might be biased. Further, the node closeness measure is a function of the passenger volume on the shortest path, instead of the path with the largest passenger volume. In this sense, we might ignore some informative paths that allow the infection spread, and thus the result should be carefully examined. Finally, although we modeled the impact of travel restrictions on the spread of COVID-19 across Japan, it should be noted that a significant portion of Japan’s population lives in a small, densely-populated area around Tokyo, and travel restrictions that benefited the rest of Japan are not sufficient to prevent widespread transmission within the metropolitan region. Thus, travel restrictions might reduce the economic damage in large parts of Japan but may still be insufficient to protect the significant population at risk within Tokyo. Further study of the role of Tokyo’s extensive public transportation network in spreading the virus within Tokyo is essential to understand how metropolitan transport closures could be used to limit transmission within Tokyo.

In summary, our study showed the impact of domestic travel restrictions on COVID-19 transmission varies by hypothetical scenarios. It suggests that domestic travel restrictions were effective to prevent the spread of COVID-19 on public transportation network. Our study highlights that, instead of strict lockdown that might seriously damage the economy, milder travel restrictions could have the similar impact on controling the domestic transmission of COVID-19 without devastating economic damage. In addition, given that Japan has not adopted strong lockdown policy to weaken the spread of the infection, our result would provide useful insight for preparing for the second or third wave without lockdown in order to balance economic and public health needs, not only in the European countries and the U.S., but also in low- and middle-income countries. Judicious application of domestic travel restrictions can serve to prevent the widespread transmission of the virus, protect fragile health systems in rural and regional areas, and buy time for these areas to prepare for the epidemic, as well as reducing the economic consequences of pandemic response. As countries reopen their economies they should consider the benefits of targeted domestic travel restrictions as a tool in their fight against COVID-19.

## Methods

### Dataset

We extracted the date of the first COVID-19 case from the Ministry of Health, Labor and Welfare website^25^ or the official website of each prefecture. Cases on the *Diamond Princess* cruise ship were excluded from this study. Other data to characterize each prefecture, including proportion of population of productive age (15–64 years old), number of companies listed with first section of the Tokyo Stock Exchange, Gross Domestic Product (GDP), and income per capita, were extracted from prefectural account data from the Japanese Cabinet Office^26^. The study was divided into three periods:

1. from January 15 (which corresponds to the date of the first COVID-19 case in Japan) to March 25, 2020 (which corresponds to the date when Tokyo prefectural governor Koike publicly announced a request to cease nonessential or/and nonurgent travel from/to other prefectures,
2. from March 25 to April 7, 2020 (which corresponds to the date when the declaration of the statement of emergency was announced progressively across prefectures^27^), and
3. from April 7 to the study end day, June 19, 2020 (which corresponds to the date when the restriction on cross-prefecture travel was removed^28^).

**Figure 3:**
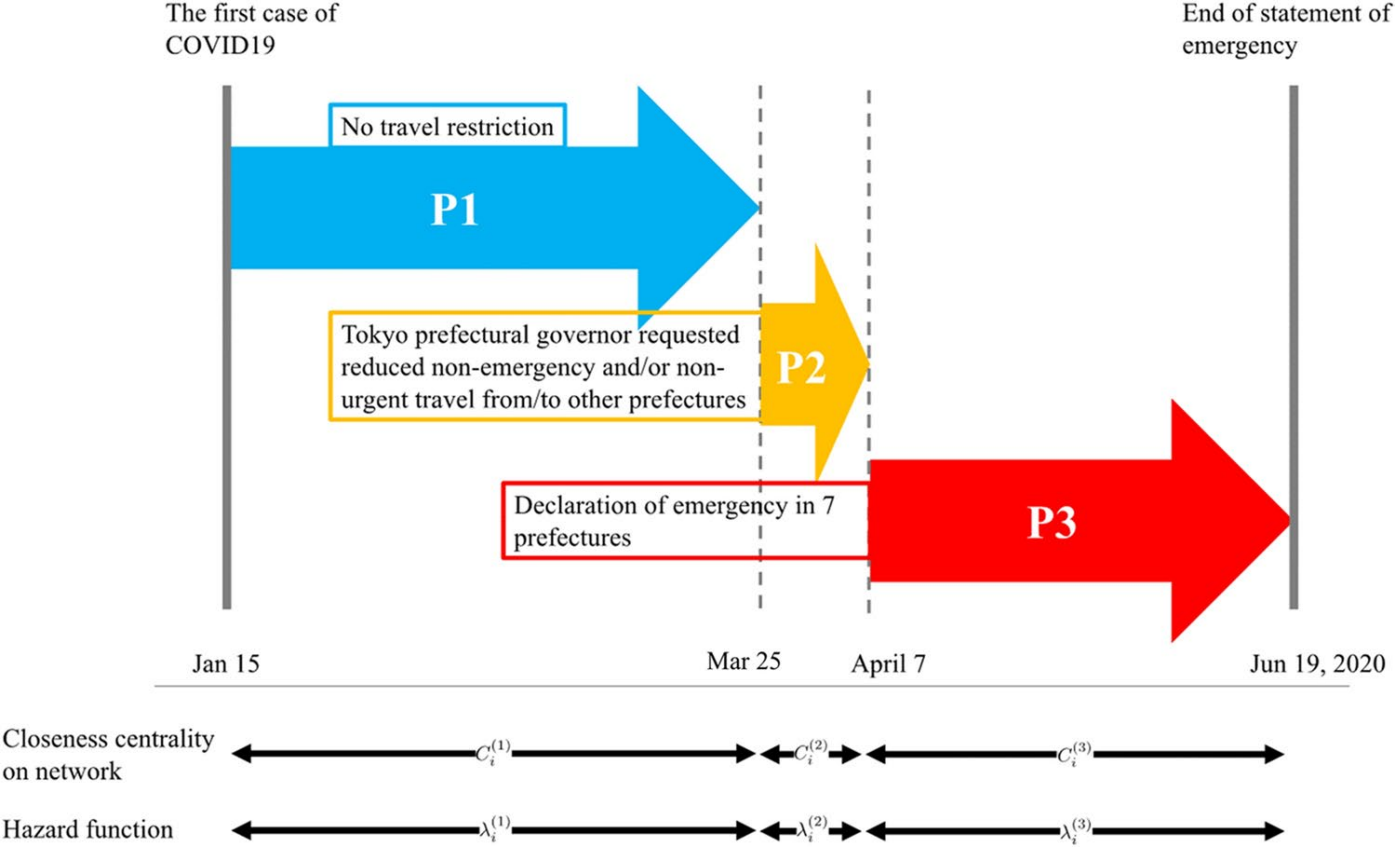
Timeline of study and associated parameters.

The study timeline is illustrated in Fig. 3.

We used passenger flow survey data conducted in 2017 by the Ministry of Land, Infrastructure, Transport and Tourism to construct the public transportation network diagram, which encompasses the passenger volumes on railways, cars, ships and airlines between two prefectures among 47 prefectures in Japan^29^. The publicly available passenger volume data are in the form of a (directed) network diagram consisting of 47 nodes (i.e., prefectures) and 1,908 edges (i.e., passenger volume from one prefecture to another). Passenger volumes less than 1,000 were excluded from the network diagram in this study. The data and *R* programs are available on request from the corresponding author.

### Closeness centrality on public transportation network

We examined the impact of restriction of public transportation networks on the transmission of COVID-19 by calculating closeness centrality from the network diagram, using the associated adjacency matrix of the network^19,30^. We calculated the closeness centrality of the *i*th prefecture on the network, defined as the inverse of the sum of the shortest distance to the other nodes:

1 $${C}_{i}=\frac{1}{{\sum }_{j=1,j\ne i}^{46}{d}_{ij}},$$

where ${d}_{ij}$ is the passenger volume on the shortest path from the *j*th to *i*th prefecture. Since we consider the risk of importation of the infection on the directed network, we focus on only the inbound paths. To take into account the fact that the network diagram and the associated centrality changed with the introduction of travel restrictions, we made the assumption that

1. the passenger volumes were not changed during P1,
2. 80% of passenger volumes from Tokyo prefecture to other prefectures were reduced during P2 due to the announcement by Tokyo prefectural governor Koike, and
3. 80% of passenger volumes between all prefectures were reduced during P3 due to the declaration of emergency.

We denote the centrality defined in (Eq. 1) under the assumption of A1, A2, and A3 as ${C}_{i}^{\left(1\right)}, {C}_{i}^{\left(2\right)}$ and ${C}_{i}^{\left(3\right)}$, respectively.

### Modeling strategy: hazard-based model with closeness centrality on public transportation network

We modeled the risk of importing COVID-19 by each municipality in Japan estimating survival probability. Let $T$ be the random variable indicating the time from the first onset date in Japan (i.e., January 15, 2020) to the first case date in the *i*th prefecture. Also define the survival probability as $F\left(t\right)=P\left(T<t\right)$ with the probability density function (pdf) $f\left(t\right)$. The hazard function for importation of COVID-19 for the *i*th prefecture is modeled as

2 $${\lambda }_{i}^{\left(j\right)}=\frac{\beta }{{C}_{i}^{\left(j\right)}}\mathrm{exp}\left({X}_{i}\alpha \right),$$

where $j=1, 2, 3$; $\beta$ is a (prefecture-common) parameter of interest, which corresponds to the parameter of the baseline hazard for all prefecture, ${X}_{i}$ is a covariate vector and $\alpha$ is a coefficient parameter vector. Variable selection was conducted based on AIC, and ${X}_{i}$ eventually included the elderly proportion of the population, the number of first section companies, GDP, and income per capita. Note that the hazard function (Eq. 2) is time invariant for each period $j$. This formulation allows the median time of importation to be proportional to the centrality ${C}_{i}$, which is consistent with Shi et al.^7^, Brockmann and Helbing^31^ and Otsuki and Nishiura^3^. Given the hazard function (Eq. 2), the pdf of survival time can be modeled as

3 $${f}_{i}^{\left(j\right)}\left(t\right)={\lambda }_{i}^{\left(j\right)}\mathrm{exp}\left(-{\int }_{0}^{t}{\lambda }_{i}^{\left(j\right)}ds\right)= {\lambda }_{i}^{\left(j\right)}\mathrm{exp}\left(-{\lambda }_{i}^{\left(j\right)}t\right).$$

A total of 47 prefectures were categorized into four separate groups:

(A) prefectures that imported COVID-19 before Tokyo prefectural governor Koike’s announcement (i.e., during P1),
(B) prefectures that imported COVID-19 between Tokyo prefectural governor Koike’s announcement and the declaration of the statement of emergency (i.e., during P2),
(C) prefectures that imported COVID-19 between the declaration of the statement of emergency and the end of study (i.e., during P4), and
(D) prefectures that did not import any COVID-19 cases until the end of study.

The likelihood of (A) is given by

4 $${L}_{A}=\prod_{i\in A}{\lambda }_{i}^{\left(1\right)}\mathrm{exp}\left(-{\lambda }_{i}^{\left(1\right)}{t}_{i}\right),$$

where ${\lambda }_{i}^{\left(1\right)}$ indicates the hazard function (Eq. 2) calculated based on the centrality during P1, ${C}_{i}^{\left(1\right)}$, and ${t}_{i}$ is days between the study start (i.e., January 15, 2020) to the date of the first COVID-19 case in the *i*th prefecture. Similarly, the likelihood of (B) is given by

5 $${L}_{B}=\prod_{i\in B}{\lambda }_{i}^{\left(2\right)}\mathrm{exp}\left\{-{\lambda }_{i}^{\left(2\right)}\left({t}_{i}-{t}_{a}\right)\right\}\mathrm{exp}\left(-{\lambda }_{i}^{\left(1\right)}{t}_{a}\right),$$

where ${\lambda }_{i}^{\left(2\right)}$ indicates the hazard function (Eq. 2) calculated based on the centrality during P2, ${C}_{i}^{\left(2\right)}$, and ${t}_{a}$ is March 25, 2020, the day on which Tokyo prefectural governor Koike requested nonessential or/and nonurgent travel from/to other prefectures. ${L}_{B}$ indicates the joint likelihood of the probability of avoiding the importation of COVID-19 for ${t}_{a}$ days before March 25 and the probability of importing COVID-19 during ${t}_{i}-{t}_{a}$. The likelihood of (C) is given by

6 $${L}_{c}=\prod_{i\in c}{\lambda }_{i}^{\left(3\right)}\mathrm{exp}\left\{-{\lambda }_{i}^{\left(3\right)}\left({t}_{i}-{t}_{b}\right)\right\}\mathrm{exp}\left\{-{\lambda }_{i}^{\left(2\right)}\left({t}_{b}-{t}_{a}\right)\right\}\mathrm{exp}\left\{-{\lambda }_{i}^{\left(1\right)}\left({t}_{a}\right)\right\},$$

where ${\lambda }_{i}^{\left(3\right)}$ indicates the hazard function (Eq. 2) calculated based on the centrality during P3, ${C}_{i}^{\left(3\right)}$, and ${t}_{b}$ is April 7, 2020, the day of the declaration of the statement of emergency. ${L}_{C}$ indicates the joint likelihood of the probability of avoiding the importation of COVID-19 for ${t}_{a}$ days before March 25, and ${t}_{b}-{t}_{a}$ days during March 25 to April 7, respectively, and the probability of importing COVID-19 during ${t}_{i}-{t}_{b}$. Lastly, the likelihood of (D) is given by

7 $${L}_{D}=\prod_{i\in D}\mathrm{exp}\left\{-{\lambda }_{i}^{\left(3\right)}\left({t}_{e}-{t}_{b}\right)\right\}\mathrm{exp}\left\{-{\lambda }_{i}^{\left(2\right)}\left({t}_{b}-{t}_{a}\right)\right\}\mathrm{exp}\left\{-{\lambda }_{i}^{\left(1\right)}\left({t}_{a}\right)\right\},$$

where ${t}_{e}$ is the end of this study period (i.e., June 19, 2020). This is the joint likelihood of the probability of avoiding the importation of COVID-19 for ${t}_{a}$ days before March 25, ${t}_{b}-{t}_{a}$ days during March 25 to April 7, and ${t}_{e}-{t}_{b}$ days during April 7 to June 19, respectively. Given these likelihoods (Eq. 4–7), the total likelihood is given by

8 $$L={L}_{A}{L}_{B}{L}_{C}{L}_{D}$$

and the maximum likelihood method was used to estimate $\alpha$ and $\beta$, which are defined in equation (Eq. 2).

### Estimate of relative risk reduction: Hypothetical scenarios approach

The effect of domestic travel restrictions on risk of importing COVID-19 in each prefecture was calculated based on the cumulative risk by comparing observed and hypothetical scenarios. Since the hazard is fixed over the time period, the cumulative risk of importation for the *i*th prefecture at the end of study date in the Observed scenario is given by

9 $${R}_{i}^{O}=1-\mathrm{exp}\left\{-{\lambda }_{i}^{\left(3\right)}\left({t}_{e}-{t}_{b}\right)-{\lambda }_{i}^{\left(2\right)}\left({t}_{b}-{t}_{a}\right)-{\lambda }_{i}^{\left(1\right)}\left({t}_{a}\right)\right\}.$$

In contrast, we assumed the following four hypothetical scenarios:

1. no travel restriction had taken place (i.e., ${C}_{i}^{\left(1\right)}$ is assumed to apply during P2 and P3),
2. lockdown Tokyo during P2 (i.e., ${C}_{i}^{\left(2\right)}$ is replaced with the centrality which assumed the passenger volumes between Tokyo and other prefectures were reduced to 1%, instead of A2) and the 7 other prefectures during P3 (${C}_{i}^{\left(3\right)}$ is replaced with the centrality which assumed the passenger volumes between the 7 prefectures and others were reduced to 1%, instead of A3),
3. the top 10 populous passenger volumes between prefectures are restricted during P3 (i.e., ${C}_{i}^{\left(3\right)}$ is replaced with the centrality which assumed the edges with the top 10 highest passenger volumes were removed, instead of A3), and
4. only airplane travel is restricted during P3 (i.e., ${C}_{i}^{\left(3\right)}$ is replaced with the centrality which assumed the passenger volumes only on airplanes became 0, instead of A3).

Then, the cumulative risk in (H1) is given by

10 $${R}_{i}^{{H}_{1}}=1-\mathrm{exp}\left(-{\lambda }_{i}^{\left(1\right)}{t}_{e}\right).$$

In a similar way to ${R}_{i}^{{H}_{1}}$, the cumulative risks in (H2–H4) are given by ${R}_{i}^{{H}_{h}}=1-\mathrm{exp}\left\{-{\lambda }_{i}^{\left(3,h\right)}\left({t}_{e}-{t}_{b}\right)-{\lambda }_{i}^{\left(2,h\right)}\left({t}_{b}-{t}_{a}\right)-{\lambda }_{i}^{\left(1,h\right)}\left({t}_{a}\right)\right\},$ where $h=2, 3, 4$ indicates scenarios (H2–H4), respectively, and ${\lambda }_{i}^{(j,h)}$ is the associated hazard function based on each scenario assumptions. Lastly, we estimated the relative risk reduction as $1-\frac{{R}_{i}^{{H}_{h}}}{{R}_{i}^{O}}$, where the relative risk reduction measures the proportion of risk reduction between Observed and the hypothetical scenarios.

## Supplementary Information

Supplementary Table.
