# Supplementary material for: Impact of domestic travel restrictions on transmission of COVID-19 infection using public transportation network approach
Source: Sci Rep. 2021 Feb 4;11:3109. doi: 10.1038/s41598-021-81806-3 (PMC7862278; doi:10.1038/s41598-021-81806-3)
Supplement: Supplementary file 1 — Supplementary Table. [file 41598_2021_81806_MOESM1_ESM.docx]

**Impact of domestic travel restrictions on transmission of COVID-19 infection using public transportation network approach
Keywords:** Travel, SARS-coronavirus, Infection control
**Authors:** Yayoi Murano, Ryo Ueno, Shoi Shi, Takayuki Kawashima, Yuta Tanoue, Shiori Tanaka, Shuhei Nomura, Hiromichi Shoji, Toshiaki Shimizu, Huy Nguyen, Hiroaki Miyata, Stuart Gilmour, Daisuke Yoneoka

Supplementary table: Date of first case and covariate information in each prefecture

| Prefecture | First case date | Proportion of elderly population | Number of companies listed with first section of the Tokyo Stock Exchange | GDP | Income per capita |
| --- | --- | --- | --- | --- | --- |
| HOKKAIDO | 2020/02/14 | 30.7 | 49 | 19,018,098 | 2,617 |
| AOMORI | 2020/04/07 | 31.8 | 4 | 4,580,259 | 2,558 |
| IWATE | NA | 31.9 | 6 | 4,674,256 | 2,737 |
| MIYAGI | 2020/03/26 | 27.2 | 23 | 9,475,481 | 2,926 |
| AKITA | 2020/03/06 | 35.6 | 4 | 3,451,335 | 2,553 |
| YAMAGATA | 2020/03/31 | 32.2 | 8 | 4,039,808 | 2,758 |
| FUKUSHIMA | 2020/03/31 | 30.2 | 13 | 7,917,871 | 3,005 |
| IBARAKI | 2020/03/22 | 28.3 | 14 | 13,056,738 | 3,116 |
| TOCHIGI | 2020/03/05 | 27.4 | 18 | 8,958,397 | 3,318 |
| GUNMA | 2020/03/07 | 28.9 | 24 | 8,528,499 | 3,098 |
| SAITAMA | 2020/03/05 | 26 | 75 | 22,689,675 | 2,958 |
| CHIBA | 2020/01/31 | 27.1 | 49 | 20,391,622 | 3,020 |
| TOKYO | 2020/02/13 | 23 | 1,777 | 104,470,026 | 5,348 |
| KANAGAWA | 2020/02/13 | 24.8 | 179 | 34,609,343 | 3,180 |
| NIIGATA | 2020/02/29 | 31.3 | 38 | 8,883,972 | 2,826 |
| TOYAMA | 2020/03/30 | 31.6 | 25 | 4,566,284 | 3,295 |
| ISHIKAWA | 2020/02/21 | 28.8 | 26 | 4,623,028 | 2,908 |
| FUKUI | 2020/03/18 | 29.8 | 15 | 3,211,131 | 3,157 |
| YAMANASHI | 2020/03/06 | 29.8 | 10 | 3,365,637 | 2,873 |
| NAGANO | 2020/02/25 | 31.1 | 38 | 8,272,256 | 2,882 |
| GIFU | 2020/02/26 | 29.3 | 28 | 7,621,798 | 2,803 |
| SHIZUOKA | 2020/03/10 | 29.1 | 53 | 17,044,389 | 3,300 |
| AICHI | 2020/02/16 | 24.6 | 222 | 39,409,405 | 3,633 |
| MIE | 2020/03/10 | 29 | 19 | 8,220,907 | 3,155 |
| SHIGA | 2020/03/05 | 25.3 | 9 | 6,381,694 | 3,181 |
| KYOTO | 2020/02/04 | 28.6 | 71 | 10,487,555 | 2,926 |
| OSAKA | 2020/02/26 | 27.2 | 423 | 38,994,994 | 3,056 |
| HYOGO | 2020/03/01 | 28.3 | 120 | 20,937,780 | 2,896 |
| NARA | 2020/03/06 | 30.3 | 6 | 3,650,718 | 2,522 |
| WAKAYAMA | 2020/02/13 | 32.2 | 8 | 3,676,471 | 2,949 |
| TOTTORI | 2020/04/10 | 31 | 4 | 1,864,072 | 2,407 |
| SHIMANE | 2020/04/09 | 33.6 | 3 | 2,520,649 | 2,619 |
| OKAYAMA | 2020/03/28 | 29.7 | 23 | 7,681,163 | 2,732 |
| HIROSHIMA | 2020/03/07 | 28.6 | 46 | 11,944,686 | 3,068 |
| YAMAGUCHI | 2020/03/04 | 33.4 | 17 | 6,087,533 | 3,048 |
| TOKUSHIMA | 2020/03/31 | 32.4 | 4 | 3,071,972 | 2,973 |
| KAGAWA | 2020/03/17 | 31.1 | 17 | 3,802,234 | 2,945 |
| EHIME | 2020/03/02 | 32.1 | 13 | 5,074,178 | 2,656 |
| KOCHI | 2020/02/29 | 34.2 | 7 | 2,419,434 | 2,567 |
| FUKUOKA | 2020/02/20 | 27.1 | 82 | 19,144,020 | 2,800 |
| SAGA | 2020/03/31 | 29.2 | 6 | 2,851,913 | 2,509 |
| NAGASAKI | 2020/03/14 | 31.3 | 4 | 4,566,162 | 2,519 |
| KUMAMOTO | 2020/02/22 | 30.1 | 5 | 5,927,626 | 2,517 |
| OITA | 2020/03/03 | 31.8 | 10 | 4,353,384 | 2,605 |
| MIYAZAKI | 2020/03/17 | 31.1 | 4 | 3,683,966 | 2,407 |
| KAGOSHIMA | 2020/04/01 | 30.8 | 11 | 5,381,809 | 2,414 |
| OKINAWA | 2020/02/14 | 21 | 5 | 4,281,963 | 2,273 |
